# Embedding the Community and Individuals in Disease Prevention

**DOI:** 10.3389/fmed.2022.826776

**Published:** 2022-04-04

**Authors:** Martine M. Bellanger, Ke Zhou, Sophie A. Lelièvre

**Affiliations:** Scientific Direction for Translational Research, Integrated Center for Oncology (ICO), Angers, France

**Keywords:** non-communicable disease, primary prevention, environment, primary healthcare, risk reduction, breast cancer, epigenome, social determinant

## Abstract

The primary prevention of non-communicable diseases is one of the most challenging and exciting aspects of medicine and primary care this century. For cancer, it is an urgent matter in light of the increasing burden of the disease among younger people and the higher frequency of more aggressive forms of the disease for all ages. Most chronic disorders result from the influence of the environment on the expression of genes within an individual. The environment at-large encompasses lifestyle (including nutrition), and chemical/physical and social exposures. In cancer, the interaction between the (epi)genetic makeup of an individual and a multiplicity of environmental risk and protecting factors is considered key to disease onset. Thus, like for precision therapy developed for patients, personalized or precision prevention is envisioned for individuals at risk. Prevention means identifying people at higher risk and intervening to reduce the risk. It requires biological markers of risk and non-aggressive preventive actions for the individual, but it also involves acting on the environment and the community. Social scientists are considering micro (individual/family), meso (community), and macro (country population) levels of care to illustrate that problems and solutions exist on different scales. Ideally, the design of interventions in prevention should integrate all these levels. In this perspective article, using the example of breast cancer, we are discussing challenges and possible solutions for a multidisciplinary community of scientists, primary health care practitioners and citizens to develop a holistic approach of primary prevention, keeping in mind equitable access to care.

## Introduction

**FIGURE 1:**
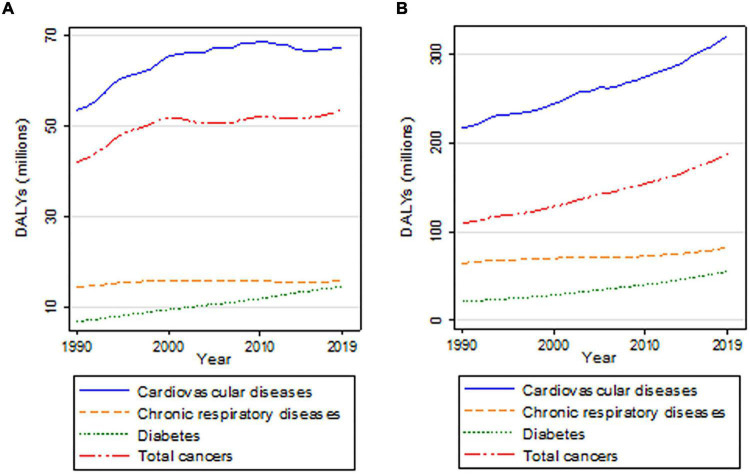
Trends in DALYs for the four major NCDs, for people aged 15–49 years **(A)** and for people aged 50 years and above **(B)**. Source: Institute for Health Metrics and Evaluation (3).

Progress has been made globally to improve the health of populations. However, non-communicable diseases (NCDs) continue to impose a burden on individuals and communities as well as on economies in countries of all income levels. According to the World Health Organization (WHO), more than 80% of premature deaths related to NCDs are due to cardiovascular diseases (CVD) (44%), cancers (23%), chronic respiratory diseases (CRD) (10%), and diabetes (4%) (1). In addition, NCDs may contribute to disability. Between 1990 and 2019, there was a marked decline in disability–adjusted-life-years (DALYs) rates for CVD, and to a lesser extent for CRD (2). However, diabetes related DALYs were on the rise both in younger and older populations; alarmingly, DALYs related to cancers have been increasing since 2015 in people aged 15–49 (Figure 1).

In 2019, the global burden of cancer in adolescents and young adults (AYA), encompassing populations aged 15–39, was 1.2 million incident cancer cases and 0.4 million cancer-related deaths, which caused 23.5 million DALYs lost (3). Western Europe was the region with the highest age-standardized incidence rate of AYA cancers in the world (75.3 for women and 67.4 for men per 100,000 person-years) (3). Strikingly, for some cancers (i.e., breast, lung, and thyroid), the burden of incidence has been shifting from advanced-aged populations to AYA for whom it might represent the most significant increase in coming years (4–6). Particularly worrisome, breast cancer mortality among AYA has ceased decreasing because of the increasing proportion of “distant diseases” (5, 7). Thus, not only cancer is more frequent in AYA, but also certain forms of the disease are very aggressive. There is a sense of urgency to harness prevention of NCDs and notably, cancers.

Prevention of the first onset of a disease in an individual (i.e., primary prevention) is one of the three pillars of medicine, along with detection and treatment. Preventing NCDs requires to know individual and environmental factors contributing to disease emergence and develop the means to act. Policies focused on “removing the cause,” as it is case with tobacco, become complex to establish when many NCDs are triggered by a combination of factors. Communication is currently centered on maintaining health with statements related to exercise and nutrition, and possibly lifestyle; but messages mostly target the general population. Epidemiology is at the forefront of primary prevention by proposing links between specific diseases and potential individual, social and environmental factors. However, the development of preventive interventions will require knowing the mechanisms that translate risk factors at the cellular level.

Individual factors contributing to NCDs may be anatomical and functional, and can often be related to genetic and epigenetic alterations (8). The epigenetic nature of NCDs directly links individuals to the lifelong impact of their environment. In health, the environment may be defined as any external factor that has an impact on an organism. Environmental risk factors may trigger global as well as specific biochemical alterations of the DNA and histone proteins that constitute the chromatin (9). Environmental elements acting on the epigenome may be categorized as modifiable risk factors, firstly because they might be changed via external actions (e.g., behavior, policies), but also because the malleable nature of the epigenome renders their epigenetic impact reversible.

The environment to which an individual is exposed during lifespan is complex. The exposome includes chemical and physical factors, nutrition and socioeconomic conditions (10). If chemical and physical factors are detrimental to the organism, they are considered pollutants. These factors have been involved in neurological disorders (11, 12), CVDs (13), respiratory disorders (14), thyroid diseases (15), allergies (16), diabetes (17), congenital disorders (18), and cancers (19). Exposure may be linked to lifestyle, but most of the time, it is imposed on people due to agricultural, industrial and urban activities. Nutrition may also increase the risk of NCDs. It may seem that risk is based on individual or family choices (20). However, food preservation and processing are also imposed on consumers (21). In contrast to other elements of the exposome, nutrition is always present and thus, it may be regarded as a positive or negative modulator of risk. Food insecurity has been linked with a risk of CVD, highlighting the importance of considering socioeconomic aspects of the environment (22).

Socioeconomic factors have an impact on NCDs globally, locally and individually (23). The NCDs unevenly affect countries depending on income level. According to the 2019 WHO global burden of disease, more than three quarters of NCD deaths occur in low-and middle-income countries (LMICs) and mostly in people 30–69 years old (1). For instance, in the poorest countries, women younger than 50 bear a higher burden of breast cancer mortality (24). Within a given country, the socioeconomically disadvantaged children and adults often suffer disproportionally from NCDs due to cumulative exposures to detrimental factors in their living areas and social stressors that prevent them from adopting a healthy lifestyle (25, 26). New evidence of the impact of the “*biological embodiment of social disadvantage*” on NCDs, beyond behavioral risk factors, has been brought based on large cohort studies in different countries and contexts (27).

Understanding the mechanisms or the causal pathways leading to the onset of NCDs is required to implement effective prevention strategies that will rely on clear connections between cumulative risks, social determinants and the body (25). In this perspective article, we report some of the most commonly used methods for primary prevention, before proposing an ecological model or a holistic approach inclusive of a multidisciplinary community of researchers, citizens, and healthcare practitioners to design interactive risk reduction programs.

## Shedding Light on Some Primary Prevention Strategies

Prevention strategies and policy-based interventions for NCDs have mainly targeted modifiable behavioral risk factors. Most strategies are nationwide or regional. Plans including taxation and smoking ban in public places along with education campaigns and targeted programs for populations of low socioeconomic status, have helped improve population health, especially in high- and upper-middle-income countries (HUMICs). There is now evidence of a decrease in mortality from lung cancer and other NCDs associated with smoking cessation (28). The 2013 WHO 25 × 25 Global Action Plan to reduce NCD-related premature mortality by 25% by 2025 aims to strengthen health systems and reinforce prevention policies for a decrease in incidence of the top four NCDs (CVD, cancers, CRD, and diabetes). This plan recommends efforts to be placed worldwide on risk factors, such as “tobacco use, physical inactivity, unhealthy diet, and harmful use of alcohol” (1). Priority is heightened since LMICs that have been under socioeconomic and epidemiological transitions, are facing the consequences of lifestyle changes (29) while the burden of infection-related diseases remains high (23).

Following the WHO recommendations on “fiscal policies for diet and prevention of NCDs,” a growing number of countries have introduced taxations on sugar-sweetened beverages. These policies influence households and individuals’ revenue and consumption while acting as potential levers to reduce diet-related risk factors (30). Implemented in few countries, like Denmark, economic incentives, such as selective taxations on foods to favor fruits, vegetables, and fiber consumption and decrease fat and saturated fat intake were effective to alter dietary behavior, leading to a reduction of the burden of ischemic heart disease and stroke, and to a lesser extent colorectal cancer (31). Fiscal policies such as tax on junk food and subsidy on fruit and vegetables in New Zealand resulted in larger health gain estimates than the 10% projected annual increase in tobacco tax over 15 years, with similar outcomes to those resulting from alcohol taxes (32). However, despite overall progress in prevention of NCDs through population-wide programs, disparities remain within and between countries. A study performed in 194 countries during the last five years reveals a huge gap between plans with policies targeting behavioral risks, such as use of tobacco, alcohol and unhealthy food, and actual implementations, particularly in countries where corporate interests may be in conflict with the health of their populations (33, 34).

Prevention through vaccination programs appear to be effective for human papillomavirus (HPV). Most of the HUMICs that allocated resources to such programs have observed a reduction in cervical cancer risk. The WHO identifies HPV and screening of women aged 30–49 as “best buys,” i.e., effective prevention interventions with cost-effectiveness ratio ≤ $_*International*_ 100 per DALY averted in LMICs (35). This endeavor has been reinforced via the 2020 WHO global strategy to accelerate the elimination of cervical cancer as a public health problem. Importantly, successful interventions were most often associated with schools and community involvements (23).

There is recent evidence of the cost-effectiveness of interventions performed at the community level and focused on diet for breast and ovarian cancers or on physical activity for NCDs including breast cancer in high income countries (36). Yet, several countries have invested in drug therapies and surgical procedures for breast cancer risk reduction. Efforts are necessary in lifestyle-related interventions for which the impact is likely to be higher since it will reduce the risk of other NCDs.

Disease prevention may require identifying the populations in which risk is highly confined. This method called risk stratification in epidemiology is most effective if quantitative assessment or biomarkers are available. The detection of mutations and single nucleotide polymorphisms has been in place for certain disorders, notably cancers (37). However, this approach is not sufficient to identify high-risk levels. For the most prevalent breast cancer for instance, the handful of well-established germline mutations only accounts for 5–10% of cases. Additional markers are being determined based on established links between the environmental or individual factors that they represent and a higher rate of disease onset. Because most of the risk factors for NCDs have been linked to epigenetic changes, a great deal of progress in risk stratification is expected to come from the identification of markers related to epigenetic alterations and thus, gene transcription products such as miRNAs that are stable in blood (9).

Risk stratification might lead to risk assessment on a per individual basis and be viewed as a key step toward individualized prevention; however, biomarkers would need to bring sufficient discrimination between risk levels (38). Individualized prevention should consider not only the level of risk of an individual but also the nature of exposure. This concept is based on findings from mechanistic investigations revealing that different types of epigenetic pathways or modifications are affected depending on the nature of the risk (9). Additionally, the method used to prevent risk-mediated epigenetic modifications needs to target the epigenetic pathway that was specifically modified by risk exposure, as shown for instance for breast tumor onset (39). To act at the individual or micro level, lifestyle and nutritional changes might be envisioned, as well as therapies. Some epigenetic changes might be temporary and easily modified, by simply removing exposure. If exposure is strong enough or long enough, epigenetic modifications might ultimately become “permanent.” Such situation would call for the use of targeted epigenetic modifiers, the nature of which remains to be clearly defined since the notion of benefit/risk is particularly stringent when treating people who have not yet developed the targeted disease.

Primary prevention cannot solely be performed at the individual level. The multifactorial risk stratification that is necessary to identify individuals at highest risk has shifted personalized prevention toward precision prevention that encompasses large numbers of participants to identify meaningful combinations of factors and their related biomarkers (40). Yet, there is an essential intermediate level in prevention between the individual and the population that would still empower the individual within the local community. In light of the social determinants of NCDs and the powerful effect of interventions at the community level mentioned above, the community at large or meso level is essential to include.

## Integration of Meso With Micro and Macro Levels in Risk Mitigation

Social and environmental factors are affecting health and well-being. Exposure occurs throughout the lifespan and at individual, community and population levels. Acting on the social determinants of health is essential to reduce the risk and burden of NCDs and relies on an integrated approach (41) involving primary healthcare. Evidence shows that the distribution of DALYs from NCDs is largely dependent on social determinants of health embedded in behavioral risk factors (1). This finding is reflected in both HUMICs and LMICs in which social inequities intertwine with behavioral and strictly environmental risk factors (e.g., air pollution and chemical exposures), usually occurring from early childhood to late adulthood (41). In its updated global action plan, WHO urges to “*strengthen and orient health systems to address the prevention and control of NCDs and the underlying social determinants through people-centered primary health care and universal health coverage*” (42).

Integrated approaches at the community and primary health care levels can stimulate synergies in service delivery and help identify individuals and groups at highest risk, before delivering interventions to mitigate risks (43). Prevention and care must go hand-in-hand, especially for family and primary care (23) and rely on the micro-, meso-, and macro-level framework. Broadly speaking, this framework refers to interactions with individuals, primary care organization for the community and national, federal and local policies, depending upon systems and targeted populations. Embedding integrated actions across different levels and across sectors as a consistent “social determinant” approach to mitigate the risk of NCDs requires the necessary policies to align priorities across sectors and to define the assessment of key outcomes. In addition, building an integrated approach of services and interventions that profiles the risks of NCDs has several advantages. While supporting individuals as essential players of their health and well-being, it empowers them to have broader impacts by involving their community and endorsing key messages otherwise hindered in large prevention campaigns or inaccessible. In return, the community is associated with other stakeholders who are able to improve conditions across the life course, and ultimately reduce social inequalities (41). This approach entails working in close partnership with public and private sectors outside the healthcare system, such as schools and colleges, food industries and environmental agencies, toward a sustainable community. Effective public health policies implemented where people live and work help tackling the root of inequality that people face in the risks of NCDs. Outcomes will be new prevention priorities, as we illustrate below with the cancer prevention strategy for AYA.

Conventional passive cancer detection (44) should be shifted toward an active targeting of modifiable environmental risk factors during windows of susceptibility occurring in young age (45). The challenge of shifting to this new approach with AYA is undeniable. The conventional approach requires working and investing within the health system. However, to target environmental risks, holistic solutions with meso- and macro-level efforts are necessary to develop effective interventions that bear sustained coverage (46, 47).

The AYA population requires new preventive strategies. For example, conventional prevention strategies for breast cancer concentrate on middle-aged and older people and rely on the early detection of incident cases, which has led to a lack of priority to focus on primary prevention to decrease breast cancer risk. This is not an effective choice for AYA due to the poor sensitivity and specificity of screening techniques among young women (44). Accumulating evidence indicates that risk is often established during windows of susceptibility and that it might then take two decades prior disease diagnosis (46). There is room to act on environmental risk exposure; lifestyle, nutrition and chemical substances have become important targets for prevention efforts (45, 48).

Community engagement is emerging as a central node in identifying and reducing the risk of NCDs. Epidemiologists and clinicians know the importance of working with the community already with patients who participate in clinical research on detection and treatment. For primary prevention research, the strong impact of the environment on disease onset has compelled investigators to define communities based on their way of life or geographical situation. A study that focused on women with a metabolic syndrome, a strong breast cancer risk factor linked to overweight and obesity, using anthropometric measurement of the breast revealed an impact of metformin in reducing adiposity (49). By focusing on a community with a metabolic risk factor for breast cancer, this work provides initial clinical evidence that more research is worth investing to further understand the mechanism of action of metformin in risk reduction. Research outcomes resulting from community-oriented investigations instead of large population cohorts are likely to rapidly benefit individuals within a community and possibly influence public policies.

Other approaches might include citizen science that bridges scientists and the public through collaborative scientific enquiry. Australian women were asked to participate in data collection to capture and classify images of alcohol advertising in a breast cancer prevention project. According to the authors, the combination of datasets from the scientists and the population brought higher levels of validity compared to existing literature solely based on investigator-driven data collection (50).

**FIGURE 2:**
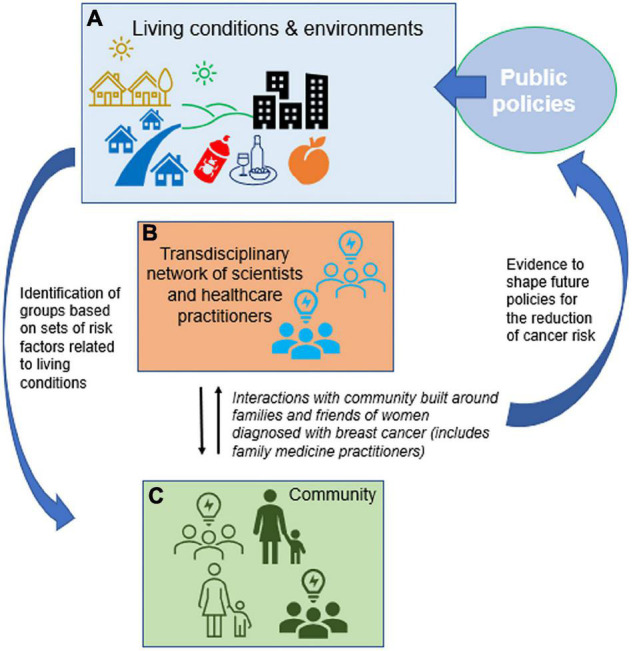
Building communities for research on primary prevention of breast cancer. **(A)** Public health policies shape most of our environment by regulating pollutants, nutrition and the recreational use of potentially harmful substances, in addition to healthcare access and delivery. Along with lifestyle and social determinants of health, chemical and physical environmental factors influence the risk of developing breast cancer. **(B)** The transdisciplinary network of IBCN scientists and healthcare practitioners aims to recruit cancer free people (friends, family members) from within the community of women who develop breast cancer to run projects focused on communities representing different sets of risk factors **(C)**. Outcomes are expected to be the design of interventions to reduce breast cancer risk and an impact on public policies.

The community is waking up to the fact that prevention of most NCDs lacks progress and people’s involvement is necessary to speed up research development and implementation. The California Breast Cancer Research Program (CBCRP) initiated a first phase called “Californians Linking Action with Science for Prevention of Breast Cancer” with the aim to build coalitions and capacity through community engagement (51). This engagement is primarily in community-based participatory research since research in primary prevention is missing. The other focus of this program is research training via dissemination and implementation. This goal is similar to that of the International Breast Cancer and Nutrition (IBCN) network that aims to develop research on the link between environmental exposure and the epigenome of the breast and propose interventions to reduce cancer risk (52) (Figure 2). Here, engagement from the community will come from women who have had breast cancer and wish to help create groups from their family and friends to participate in research. For each project, scientists will define the environmental exposures to be studied and age range for cancer risk in order to target the community belonging to defined risk categories. Education on scientific aspects of the proposed research will be essential to entail informed decision to participate from the community (53).

Transdisciplinary efforts in primary prevention with participation from the community require policy plans for funding focused on communities that are particularly at risk for the onset of NCDs. These plans may be at the regional level in a particular country, like the CBCRP funding program for which phase 2 of funding support is on risk factors of “*disadvantaged, high risk communities with unmet social needs*” (54). On a global level, the European Union (EU) has made NCDs a main part of their action plans for health. Some of their Horizon Europe funding calls focus on adolescents in light of the report from the Lancet commission on adolescent health and well-being (55). This effort is paramount since it will enable scientists to work with a population that represents a main window of susceptibility to risk factors for NCDs, as it is the case for breast cancer for instance. Adolescents are particularly vulnerable for the setting of NCDs later in life both from biological and behavioral standpoints. Indeed, this period of life is highly malleable epigenetically and for establishing durable lifestyles and habits.

Research advocates from the community should be present from the start of new projects to help strategize and alleviate potential obstacles inherent to developing research with a group that is free of the disease being targeted. However, as highlighted in a thoughtful study of the strategies for genetic testing linked to cancer risk, new investigations on the ethical consequences of primary prevention research are warranted (56).

## Conclusion

Scientists and health care practitioners are pillars to accompany and guide a community of individuals involved in risk reduction. This community is likely to encompass families since windows of susceptibility are prominent during childhood. Family medicine will have to be integrated in such efforts. One possible direction to involve the community of family medicine practitioners is via medical education programs and participation in translational research networks.

Our research perspective supports the core concept of community and individuals’ engagement in the process of research, from its inception, and the process of using research findings to design NCD risk reduction programs. The foundations of our promising approach have their roots in the capability of the community members to define their healthcare and prevention commons and manage them collaboratively to create their own favorable individual and collective conditions of healthy environment.

## Data Availability Statement

The original contributions presented in the study are included in the article/supplementary material, further inquiries can be directed to the corresponding author/s.

## Author Contributions

SL and MB wrote and edited the manuscript. KZ participated in writing and editing the manuscript, and prepared Figure 1. All authors contributed to the article and approved the submitted version.

## Conflict of Interest

The authors declare that the research was conducted in the absence of any commercial or financial relationships that could be construed as a potential conflict of interest.

## Publisher’s Note

All claims expressed in this article are solely those of the authors and do not necessarily represent those of their affiliated organizations, or those of the publisher, the editors and the reviewers. Any product that may be evaluated in this article, or claim that may be made by its manufacturer, is not guaranteed or endorsed by the publisher.
